# Peripheral vision in real-world tasks: A systematic review

**DOI:** 10.3758/s13423-022-02117-w

**Published:** 2022-05-17

**Authors:** Christian Vater, Benjamin Wolfe, Ruth Rosenholtz

**Affiliations:** 1 Institute of Sport Science, University of Bern, Bremgartenstrasse 145, CH-3012 Bern, Switzerland; 2 Department of Psychology, University of Toronto Mississauga, Toronto, Canada; 3 Computer Science and Artificial Intelligence Laboratory, Massachusetts Institute of Technology, Cambridge, MA USA; 4 Department of Brain and Cognitive Sciences, Massachusetts Institute of Technology, Cambridge, MA USA

**Keywords:** Peripheral vision, Walking, Aviation, Driving, Sports science

## Abstract

Peripheral vision is fundamental for many real-world tasks, including walking, driving, and aviation. Nonetheless, there has been no effort to connect these applied literatures to research in peripheral vision in basic vision science or sports science. To close this gap, we analyzed 60 relevant papers, chosen according to objective criteria. Applied research, with its real-world time constraints, complex stimuli, and performance measures, reveals new functions of peripheral vision. Peripheral vision is used to monitor the environment (e.g., road edges, traffic signs, or malfunctioning lights), in ways that differ from basic research. Applied research uncovers new actions that one can perform solely with peripheral vision (e.g., steering a car, climbing stairs). An important use of peripheral vision is that it helps compare the position of one’s body/vehicle to objects in the world. In addition, many real-world tasks require multitasking, and the fact that peripheral vision provides degraded but useful information means that tradeoffs are common in deciding whether to use peripheral vision or move one’s eyes. These tradeoffs are strongly influenced by factors like expertise, age, distraction, emotional state, task importance, and what the observer already knows. These tradeoffs make it hard to infer from eye movements alone what information is gathered from peripheral vision and what tasks we can do without it. Finally, we recommend three ways in which basic, sport, and applied science can benefit each other’s methodology, furthering our understanding of peripheral vision more generally.

## Introduction

**Fig. 1:**
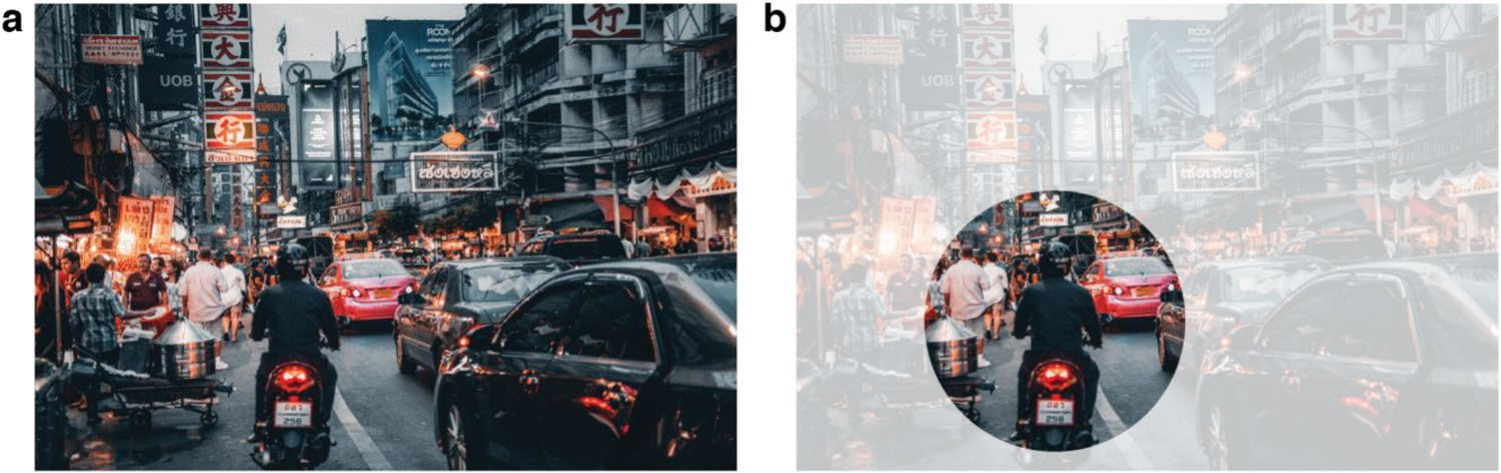
Illustration of an urban street scene (“Crowded Street With Cars Passing By”, by Suzukii Xingfu; sourced from Pexels.com, under CC0) with cars, motorbikes, and pedestrians; (**a**) shows the entire scene, (**b**) shows a visualization of a useful field, approximately 15° radial from fixation, illustrating a commonly held misconception of the region of visual space around the point of fixation in which observers can perceive visual information, with the surrounding region faded out to illustrate how much information is missing

Peripheral vision, the visual field beyond our current point of gaze (i.e., outside the parafovea or the central 4–5° around the fovea; Larson & Loschky, 2009), provides information that is essential for a vast range of tasks in everyday life. For example, walking and driving require us to be aware of the behavior of others so as not to collide with them (see Fig. 1 for a driving example). It is impossible to always fixate the most relevant visual information at the right time; our environment sometimes changes in an unpredictable manner, and the relevant information may not be localized to a single location. That peripheral vision is vital to our everyday life also becomes apparent from clinical cases of its absence. Patients suffering from retinitis pigmentosa, a disease that progressively robs the patient of peripheral input, have profound difficulties navigating the world, since so much happens outside their field of view (Crone, 1977; Pagon, 1988).

What we can or cannot do with peripheral vision has mostly been studied in fundamental research rather than applied research. This work has shown that we acquire information from the entire visual field when the task requires it, as when perceiving the gist of a scene (Boucart et al., 2013; Ehinger & Rosenholtz, 2016; Geuzebroek & van den Berg, 2018; Larson et al., 2014; Larson & Loschky, 2009; Loschky et al., 2019; Trouilloud et al., 2020; Wang & Cottrell, 2017). In fact, we use peripheral input to guide search (Hulleman & Olivers, 2017), and it can help us identify objects away from fixation, even when they are present in complex environments (Wijntjes & Rosenholtz, 2018; but see Ringer et al., 2021, and Sanocki et al., 2015, for cases where identification performance was impaired). Many experiments in basic vision research place few demands on participants that would push them to use peripheral vision, inadvertantly encouraging interpretations that focus on foveal vision (a point discussed in Gegenfurtner, 2016; Rosenholtz, 2016). If, for example, you are participating in a classic visual search experiment, looking for Ts among Ls, doing this task requires you to sequentially search through the array of letters, and it is tempting to focus on the sequence, rather than on what informs the sequence of gaze shifts and how they are planned, which rely on peripheral vision. That, then, begs the question: What might happen if we did not have the luxury of focusing on a single task at a time, which we seldom can in life outside the laboratory?

Our goal in this review was to discuss how peripheral vision is used in driving, walking, and aviation tasks, where successfully using it is necessary to our ability to complete these tasks. This builds on our previous work on peripheral vision in a range of contexts, from basic vision science (Rosenholtz, 2016, 2020) to driving (B. Wolfe et al., 2017; B. Wolfe et al., 2020) to sports (Vater et al., 2020). Because the task demands inherent to walking, driving, and aviation draw on the same fundamental processes and attributes that we have discussed in our previous work, our goal here is to extend this prior work and to identify how peripheral vision is used in a trio of very different real-world activities. Our goal is to not only see how peripheral vision is used in these applications, but to spur future research in both applied and basic areas to deepen our understanding of peripheral vision.

To provide context for our discussion of peripheral vision, we first provide a brief orientation in peripheral vision in basic vision science research, focused on the mechanisms of visual perception. Second, we discuss how peripheral vision is used in sports, and how the very different visual demands of a sport push players to adopt strategies that are not seen in simple laboratory experiments. Together, these brief reviews serve to set the stage for the present review, and contextualize our conclusions. We then dive into the topic of this review in earnest, examining how peripheral vision is used in tasks where it is an integral component and what impacts our ability to use peripheral vision. Finally, we conclude by discussing how our understanding of peripheral vision has been enhanced by this exercise, and provide three suggestions regarding future peripheral vision research and the information required for different tasks that we hope will foster new and innovative research.

## The basics of peripheral vision

In order to understand why peripheral vision is different from foveal vision, we need to start with anatomy. The fovea, the location on the retina where light is focused, is the area of highest photoreceptor density and comprises 1% or less of the total surface of the retina, but accounts for 50% of visual cortex (Curcio et al., 1990; Tootell et al., 1982). Given this anatomical bias, peripheral visual input must be represented differently than foveal input, and phenomenologically, we notice that we are less able to resolve fine detail in the periphery (Anstis, 1974; Strasburger et al., 2011), that we have slightly poorer color vision (Abramov & Gordon, 1977; Gordon & Abramov, 1977; Hansen et al., 2009), and that, in general, our experience of vision away from our point of gaze is quite different (for a review, see Rosenholtz, 2016), even if we do not think about it much (Rosenholtz, 2020).

How, then, do the differences between foveal and peripheral vision impact our perceptual experience and abilities? Perhaps the most noticeable of these impacts is the phenomenon of visual crowding (Bouma, 1970), where objects near each other in the periphery become difficult to identify. This is not a lack of acuity or resolution, but a consequence of other differences between foveal and peripheral vision. While crowding is often studied with letters, it occurs for all objects in the periphery (e.g., letters, shapes, objects, patterns; for reviews, see Ringer et al., 2021; Rosenholtz, 2016, 2020; Sanocki et al., 2015). While it can be difficult to identify objects in the periphery because of crowding, we do not want to give the impression that the periphery is just a jumble of unrecognizable objects; the information present is useful and is used for a range of tasks.

Given the problem of crowding, one may think that peripheral vision only provides information for saccade planning, since if crowding renders peripheral objects unidentifiable, recognizing them requires making a saccade to bring them to the fovea. This is one role of peripheral vision, but only one among many. A key part of this process is covertly attending to the target of an impending saccade (i.e., by making use of peripheral vision) before the eye moves; this process of presaccadic attention (cf., Deubel & Schneider, 1996; Kowler et al., 1995) is necessary to plan accurate saccades to peripheral targets. However, the act of planning a saccade alone (i.e., without foveation of the target) can make peripherally crowded objects easier to identify and seems to access peripheral information that is otherwise inaccessible (Golomb et al., 2010; Harrison, Mattingley, & Remington, 2013a; B. Wolfe & Whitney, 2014). In fact, this peripheral information is remapped prior to the eye moving (Harrison, Retell, et al., 2013; B. Wolfe & Whitney, 2015), and is likely a key component of how we maintain a stable percept of the world in spite of making several saccades per second (Stewart et al., 2020).

In addition, even without planning a saccade, peripheral vision provides a great deal of useful information (Rosenholtz, 2016). For example, recognition of crowded objects can be improved by perceptual grouping (e.g., Banks & White, 1984; Bernard & Chung, 2011; Livne & Sagi, 2007; Manassi et al., 2012) and scene context can help resolve ambiguous peripheral information (Wijntjes & Rosenholtz, 2018). In addition, though crowding makes tasks like recognizing letters flanked by other letters difficult (but also in complex real-world scenes; cf., Ringer et al., 2021; Sanocki et al., 2015), it preserves sufficient information to support a range of tasks, for example tracking multiple objects at once (Pylyshyn & Storm, 1988) and understanding the gist of a scene at a glance (Boucart et al., 2013; Ehinger & Rosenholtz, 2016; Geuzebroek & van den Berg, 2018; Larson et al., 2014; Larson & Loschky, 2009; Loschky et al., 2019; Trouilloud et al., 2020; Wang & Cottrell, 2017). In both tasks, the distributed nature of the information needed for the task, as well as the need to keep up with temporal constraints, requires using peripheral vision. In other tasks, we do not have to look at each individual item in a group (B. Wolfe et al., 2015) to determine mean object size (Ariely, 2001) and orientation (Dakin & Watt, 1997), facial emotion (Haberman & Whitney, 2012; Yamanashi Leib et al., 2014) or the heading direction of walking figures (Sweeny et al., 2013).

For that matter, the information we can glean from peripheral vision can be impacted by attention, and the two are often considered together. At a relatively simple level, covert attention (i.e., attending to an object away from the point of gaze) can modestly improve contrast sensitivity (Cameron et al., 2002; Carrasco, 2011), processing speed (Carrasco et al., 2006), change-detection performance (Vater, 2019), and even the perception of an object (Carrasco & Barbot, 2019). There have been attempts to quantify the space around the locus of gaze within which covert attention facilitates object recognition: The functional visual field (Mackworth & Morandi, 1967), alternately known as the useful field of view (Ball et al., 1988; see also Ringer et al., 2016, for a recent UFOV (useful field of view) study using natural scenes). It should, however, be noted that highly salient stimuli (i.e., stimuli that are unusual or different to their surroundings) can be particularly easy to detect with covert attention (Itti & Koch, 2000).

In summary, basic vision science tells us that although peripheral vision might be limited, it remains useful for a number of tasks. For example, we can plan saccades, track multiple objects at once, perceive the gist of a scene or set, and perform some object-recognition tasks. These results suggest that peripheral vision is a powerful foundation on which many of our actions in daily life are constructed. It can be hard, particularly in the laboratory, to see the extent to which this is true, since many vision experiments simplify the world as much as possible, but if we step outside the laboratory, we might gain a better appreciation for how we *really* use peripheral vision.

## Peripheral vision in sports

We can learn more about use of peripheral vision by studying vision in sports. Players do not have the luxury of simple visual environments. In most sports, multitasking is required and actions must be made quickly in order to be effective. As an example, football players often look at the player with the ball and use peripheral vision to monitor other players (opponents and teammates) and to position themselves in an optimal way to prevent the opposing team from scoring a goal (Vater et al., 2019). Vater et al. (2020) provide an overview on how athletes from different sports use peripheral vision and discuss three gaze strategies they use. In some settings, a player might need to monitor multiple locations, each of which require information only available with central vision. In this situation, players adopt a *visual pivot* strategy, choosing a gaze location that minimizes the time required to move their eyes to fixate a target once the player decides which one needs fixating. However, this strategy comes with its own costs, since visual information is suppressed during a saccade, and while these intervals of suppression are brief, the lack of information can prove decisive. To avoid this, a player might adopt a *gaze anchor* strategy*,* keeping their gaze in one location and relying exclusively on peripheral vision to monitor other locations, in spite of the differences between foveal and peripheral vision.

Finally, similar to the vision science notion of the functional visual field, in the *foveal spot* strategy, players optimize their fixation to gather information from both the target of fixation and its surround. For example, in a one-on-one situation in soccer, a defender fixates the hip of the opposing player with the ball, since this provides information about the player’s direction of travel (cf. Vaeyens et al., 2007). Fixating the hip rather than, for example, the head also reduces the risk of falling for a head fake (Weigelt et al., 2017) – another reason why it is better to fixate the hip and not the head.

On the whole, the gaze strategies adopted when playing sports suggest that, in complex situations, under time pressure, we leverage peripheral vision in a way that we simply do not in the lab, although we can see echoes of laboratory behavior on the sports field. A player adopting the visual pivot strategy is using a similar approach to what research participants do in the lab when told to monitor multiple moving targets in a multiple object-tracking task (Fehd & Seiffert, 2008, 2010; Vater, Kredel, & Hossner, 2016a). A gaze anchor, where the player’s gaze stays in one spot, is not dissimilar to what participants might do with unpredictable or brief objects, or in scene gist studies, where there is simply no time to move the eye there before the stimulus vanishes. For that matter, a foveal spot strategy looks a great deal like functional visual field strategies in search (Motter & Simoni, 2008; J. M. Wolfe, 2021; Wu & Wolfe, 2018).

## Goals of the current review

Taking inspiration from discussions of peripheral vision in sports, and building on our interest in peripheral vision in a wide range of situations, we asked what everyday tasks might have unacknowledged peripheral vision components. In this paper, we review how drivers, pedestrians, and pilots use peripheral information, and which factors change our ability to use it. In doing so, we aim to elucidate patterns of behavior that indicate the use of peripheral vision and to draw connections between fundamental and applied research.

## Method

### Identification

To conduct this systematic review we followed the PRISMA (Preferred Reporting Items for Systematic Reviews and Meta-Analyses) procedure (Moher et al., 2009) and conducted a systematic literature analysis in April 2019 using Pubmed, Scopus, ScienceDirect, and Web of Knowledge. The results of each search were exported as ris- or txt-files and imported into citavi® (version 6, 2018). To identify studies, we only included peer-reviewed articles, written in English, with accessible full texts. If the databases included filters, we used them to exclude conference abstracts, dissertations, book chapters, and reviews. We defined the search terms a priori and combined them with Boolean operators (“AND”, ”OR”, “NOT”) as follows: "attention* OR peripheral*" AND “eye movement” OR “eye tracking” OR "gaze*" OR “visual search” AND "walking* OR driving* OR aviation*" NOT “sport*”. The “*” is a wildcard operator (e.g., when searching for sport*, “sports” or “sportsmen” will also be found). We searched for these terms in title, abstract and, if available, keywords (for details, see Table 1, “Identification”).

**Table 1:** Search strategy used for including papers in the reviewed set

Identification				Screening		Eligibility	
Databases searched	Inclusion criteria	Exclusion criteria	Search terms used	Abstract exclusion criteria	Example	Search *full text* for terms	Other exclusion criteria
Pubmed (title, abstract) Scopus (title, abstract, keywords) ScienceDirect (title, abstract, keywords) Web of Knowledge (title, abstract, keywords)	Peer-reviewed Full-text English language	Conference abstracts Dissertations Book chapters Reviews	"attention* OR peripheral*" AND “eye movement” OR “eye tracking” OR "gaze*" OR “visual search” AND "walking* OR driving* OR aviation*" NOT “sport*”	Diseases	Parkinson, dementia,	"peripheral" OR "covert" OR "attention"	No car driving, aviation or walking Not empirical (e.g., review) No points in evaluation scheme (see *Table* 2)
				Drugs	Alcohol; cannabis; ecstasy;		
				Fatigue	fatigue during driving; car accidents		
				Ageing	Cognitive impairments in older people		
				Radiographs	scanning radiographs		

Identification includes the databases searched, the filter criteria used and the search terms used. In the screening columns, we name the excluded topics and provide some examples. In the first eligibility column, the search terms that were used for the full text are shown. In case these search terms were not found, studies were excluded from the analyses. In the second column, we show further reasons for exclusion

### Screening

Using this search strategy, we found 975 unique articles. Of these, 850 were primarily focused on topics outside the scope of this review (e.g., diseases, drugs, fatigue, aging, and radiography (for examples, see Table 1, “Screening”)). Excluding these, we then searched the remaining 125 full texts (86 driving, 15 aviation, 24 walking) for the keywords “peripheral,” “covert,” or “attention”. If none of these search terms were found, the article was removed from the set. In addition, we manually excluded papers that did not focus on driving, aviation, or walking that were not otherwise excluded.

**Fig. 2:**
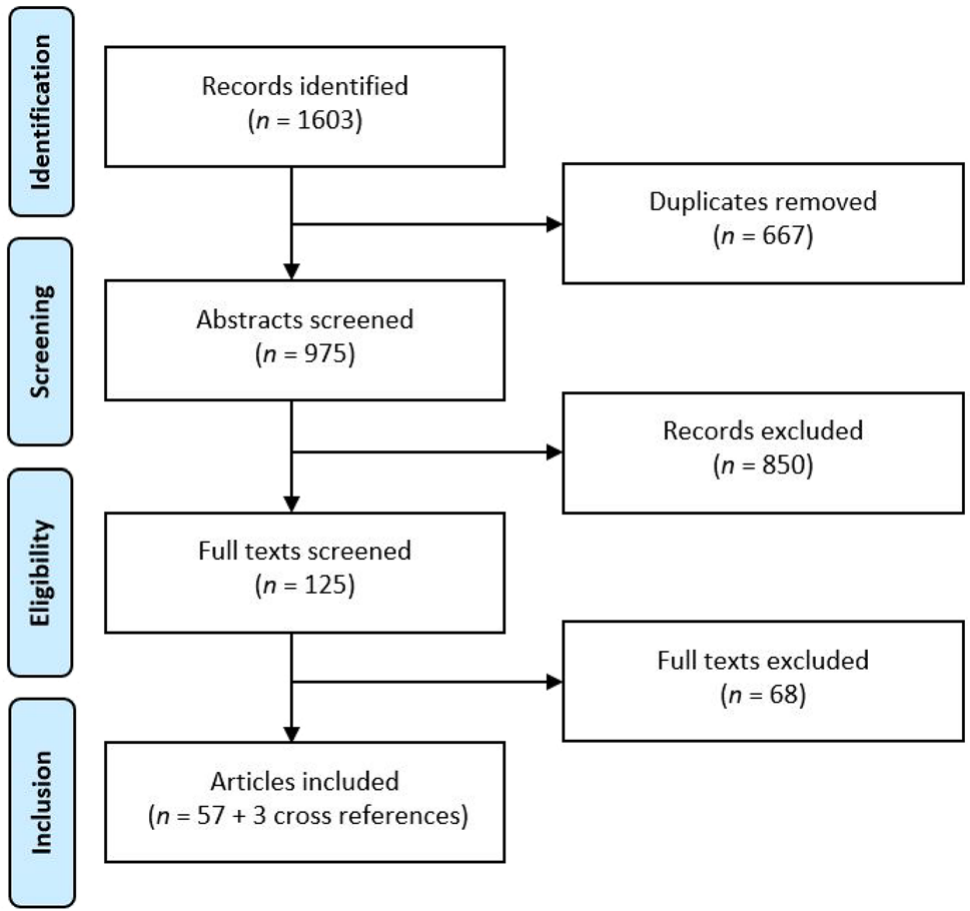
PRISMA flowchart showing the number of articles excluded and included in the different stages of the screening process. See Table 1 for inclusion and exclusion criteria

While this procedure risks missing articles that might inform our understanding of peripheral vision because they do not use the terms we required, finding such papers would require reading *all* existing papers even remotely related to the topics of this review, and potentially interpreting them in ways the authors did not, which is not possible. While imperfect, our selection procedure does enable us to have a formal process for including papers in our set, and those that focus on the role of peripheral vision are likely, in our estimation, to use the terms we searched for. We included both simulated lab experiments and real-world experiments because laboratory experiments provide important information that can be difficult to acquire from real-world experiments. It is sometimes safer to bring real-world tasks into the lab and create a controlled environment rather than on the road or in flight, especially when forcing participants to use their peripheral vision or a specific gaze pattern. In a simulator researchers can approximate the operational reality of driving a car with none of the risks to the driver or other road users. However, such simulators have their limits, since even the most high-fidelity simulation remains a simulation and there are few consequences for failure, unlike on the road. While these approaches, and other laboratory-based paradigms (e.g., screen-based environments) have the potential to reveal key elements of how and why we use peripheral input, there will always be limits to what we can learn in the lab (Godley et al., 2002), and the results will need to be validated in the real world. Based on the full text of papers, we excluded those papers that were not empirical studies (e.g., reviews) (see Table 1, “Eligibility”). In addition, while screening the full texts, we found three additional cited papers that fulfilled the inclusion criteria and added these to the set. This resulted in a final set of 60 papers (see Fig. 2).

### Quantitative analyses

Because the 60 included papers focused to different degrees on peripheral vision – their main research question may not be on peripheral vision – we developed 11 binary criteria (see Table 2) for describing papers and to help readers to identify papers that are relevant for them. We consider these 11 criteria as key points in the context of this review, but one should not interpret the result of the scoring procedure as a measure of paper quality; the score merely indicates whether the authors mentioned the topics listed. If the paper met a criterion, we scored it with a one for that criterion, if not, we scored it with a zero. For example, if a paper described inhomogeneities in the human retina or discussed visual crowding and its impact on peripheral vision and visual perception, it was noted as having “characterized visual capabilities.” As in Vater et al. (2020), we will use the term “functionality” to describe what peripheral vision is used for. The first author provided initial assessments for all studies across the 11 criteria, after which all three authors discussed the assessments for each paper until consensus was reached.

**Table 2:** Overview of the criteria used to compare reviewed papers

Introduction		Methods		Results			Discussion			
Visual capabilities characterized	Predictions on peripheral-vision usage	Peripheral-vision manipulation	Attentional manipulation	Peripheral vision manipulation check	Compares foveal and peripheral vision	Compares with limited peripheral vision	Different attentional load/demands	Discussions based on own results	Functionality discussed	Effects on actions discussed
Visual acuity Crowding Saccade properties Visual field Retina characteristics	Differences between peripheral vision conditions Effects of peripheral events on eye movements Effects of peripheral events on performance Changes in useful field of view	Eccentricity of objects Moving window	Spatial cueing to periphery Attentional/cognitive load/demands	Peripheral event detection Peripheral vision blocked	Task in fovea vs. periphery Changes in saccade behavior Eccentricity differences Dual tasks (with additional foveal task)	Occlusion of fovea or periphery Limited field of vision	Effects of secondary task High vs. low risk Visual demands increased (e.g., additional pedestrians) Cognitive workload Demands of environment (e.g., walking surface)	Discussion of reported results in paper	What is peripheral vision good for (or not)? When is it used? Why does the useful field of view change? Perceptual performance changes	Reference to actions performed Reference to motor control Effects on performance

In the second row of the table, 11 criteria are named across paper sections. In the rows below, we provide the criteria required to meet the criteria; one or more of these conditions was required to be met for the criteria to be scored with a 1 (see Table 3 for scores for each paper included)

## Quantitative results

### Study characteristics

Of the 60 included studies, six examined questions in aviation, 36 in driving, and 18 in walking. These studies investigated the use of peripheral vision in a variety of different ways, mostly in real-world situations (22 studies) or simulators (24 studies), but also head-mounted displays (three studies) or computer desktop-based paradigms (13 studies). Most of the walking studies (72%) examined peripheral vision in real-life situations (11% head-mounted display/HMD, 11% screen, 6% simulator/treadmill for the other testing modalities). In contrast, in driving, 50% of studies used a driving simulator, with the remaining studies using other modalities (26% on-road, 21% screen-based, 3% head-mounted displays). In aviation, simulators and screens were each used in 50% of the studies. There were also differences in the application of eye tracking to monitor eye movements. While eye-tracking devices were used in 79% of included studies, the three research areas used it to different extents (aviation: 100%, driving: 81%, and walking: 67%).

### Criteria

Our criteria, listed in Table 3, are a tool for categorizing whether a study discussed a functionality of peripheral vision. The last column of Table 3 shows the sum of points each study received for our pre-defined peripheral vision criteria. The studies that met most (10/11) of our criteria were the walking study by Miyasike-daSilva and McIlroy (2016) and the driving study by Gaspar et al. (2016).

**Table 3:** Overview of reviewed papers in aviation, driving, and walking (this table is available as an excel-file on the Open Science Framework at*:* https://osf.io/vea5r/?view_only=ba8597fef6514be68082d9e878fff5d2

Study characteristics					Introduction		Methods			Results			Discussion				Points
Task	First author	Year	Environment	Eye-Tracking	Visual capabilities characterized	Predictions on peripheral vision usage	Peripheral vision manipulation	Attentional manipulation	Peripheral vision manipulation check	Compares foveal and peripheral vision	Compares with limited peripheral vision	Different attentional load/demands	Discussions based on own results	Functionality discussed	Effects on actions discussed	Functionality of peripheral vision	
Aviation	Brams	2018	Screen (videos)	yes	0	0	0	0	0	0	0	0	1	1	0	Detection and “global scan” (similar to scene gist)	2
Aviation	Imbert	2014	Screen (videos)	yes	1	1	1	0	0	0	0	0	1	0	0	-	4
Aviation	Kim	2010	Simulator	yes	0	0	0	0	0	0	0	0	1	0	0	-	1
Aviation	Robinski	2013	Simulator	yes	0	0	0	0	0	0	0	0	1	0	0	-	1
Aviation	Schaudt	2002	Screen (videos)	yes	0	1	0	0	0	0	0	1	1	0	1	-	4
Aviation	Yu	2014	Simulator	yes	0	0	0	0	0	0	0	0	1	1	1	Control keys	3
Driving	Alberti.	2014	Simulator	yes	0	1	1	0	0	0	0	0	1	1	1	Speed estimation	5
Driving	Beh	1999	Screen (videos)	no	0	0	0	0	0	0	0	1	1	0	0	-	2
Driving	Bian	2010	Simulator	no	0	0	1	1	0	1	0	1	1	0	0	-	5
Driving	Briggs	2016	Screen (videos)	yes	0	0	1	0	0	1	0	0	1	1	1	Dual-tasking leads to visual and cognitive tunneling	5
Driving	Cooper	2013	Simulator	yes	0	0	0	1	1	1	0	1	0	1	0	Peripheral vision used for lane keeping	5
Driving	Crundall	2002	Screen (videos)	yes	0	1	1	0	1	1	0	1	1	0	0	-	6
Driving	Crundall	2004	Simulator	yes	0	1	1	1	0	0	0	1	0	0	0	-	4
Driving	Danno	2011	Real world, Simulator	yes	1	1	0	1	1	1	0	1	1	1	1	Peripheral preview	9
Driving	Doshi	2012	Simulator	yes	0	0	1	0	0	0	0	0	0	0	0	Covert attention attracted by peripheral event	1
Driving	Edquist	2011	Simulator	yes	0	0	0	0	0	0	0	1	0	1	0	Peripheral monitoring	2
Driving	Gaspar	2016	Simulator	yes	1	1	1	1	1	1	1	1	1	1	0	Peripheral monitoring	10
Driving	Harbluk	2007	Real world	yes	0	0	0	1	0	0	0	1	0	0	0	-	2
Driving	Huestegge	2016	Screen (single images)	yes	1	1	1	0	1	1	0	0	1	1	1	Peripheral preview	8
Driving	Janelle	1999	Simulator	yes	0	1	0	0	0	0	0	1	1	0	0	-	3
Driving	Kountouriotis	2011	Simulator	yes	0	0	1	0	1	1	0	0	1	1	1	Visual feedback of road edges	6
Driving	Kountouriotis	2016	Simulator	yes	0	0	0	1	0	0	0	1	0	1	1	Avoiding costs of saccades	4
Driving	Lamble	1999	Real world	no	0	1	1	1	0	1	0	0	1	1	1	Eccentricity costs	7
Driving	Lehtonen	2014	Real world	yes	0	1	1	0	1	1	0	0	1	1	1	Knowledge/memory (expert advantage) affects the use of peripheral vision	7
Driving	Lehtonen	2018	Real world	yes	1	1	1	0	1	1	0	0	1	1	1	Uncertainty affecting gaze transitions back to relevant information, eccentricity costs	8
Driving	Lin	2010	Simulator	yes	0	0	0	1	0	0	0	1	1	0	0	-	3
Driving	Luoma	1983	Screen (single images)	yes	1	0	0	0	0	0	0	0	1	1	0	Peripheral preview	3
Driving	Mayeur	2008	Simulator	no	1	0	1	0	0	1	0	1	1	0	0	-	5
Driving	Mourant	1970	Real world	yes	0	0	0	0	0	0	0	0	1	1	0	Monitoring and preview	2
Driving	Patten	2006	Real world	no	0	0	1	1	0	0	0	1	1	0	0	-	4
Driving	Seya	2013	Simulator	yes	1	0	1	0	1	1	0	0	1	1	0	Avoid costs of saccades	6
Driving	Shahar	2012	Screen (videos)	yes	0	0	0	0	0	0	0	0	1	1	1	Peripheral preview	3
Driving	Shinoda	2001	HMD, Simulator	yes	1	1	1	0	1	1	0	0	1	1	1	Peripheral preview (especially in situations with high probability)	8
Driving	Strayer	2003	Simulator	yes	0	0	0	1	0	0	0	1	0	1	0	Peripheral preview	3
Driving	Summala	1996	Real world	no	0	0	1	1	0	1	0	1	1	1	0	Eccentricity costs and dual-tasking costs	6
Driving	Tsai	2007	Simulator	yes	0	0	0	1	0	0	0	1	0	0	0	-	2
Driving	Underwood	2003	Real world	yes	0	0	0	1	0	0	0	1	1	1	0	Lead vehicle as "pivot"; peripheral preview	4
Driving	Underwood	2005	Screen (videos)	yes	0	1	0	1	0	0	0	1	0	0	0	-	3
Driving	Victor	2005	Simulator	yes	1	0	0	1	0	0	0	1	1	0	0	Peripheral monitoring under higher cognitive load	4
Driving	Zhang	2016	Simulator	yes	0	0	0	1	0	0	0	1	1	1	0	Anger reduces the ability to process peripheral information	4
Driving	Zhao	2014	Screen (single images)	yes	0	0	0	1	1	0	0	0	1	0	0	Distribution of attention as expertise characteristic	3
Driving	Zwahlen	1989	Real world	no	0	0	1	0	0	1	0	0	1	0	0	-	3
Walking	Bardy	1999	Screen (videos)	no	1	0	1	0	0	1	0	0	1	1	1	Functional use of optic flow	6
Walking	Berensci	2005	Screen (videos)	no	1	0	1	0	0	1	0	0	1	1	1	Reduce body sway	6
Walking	Cinelli	2009	Real world	yes	0	0	0	0	0	0	0	0	1	0	0	-	1
Walking	Feld	2019	Real world	yes	0	0	0	0	0	0	0	0	1	1	0	Monitor environment	2
Walking	Hasanzadeh	2018	Real world	yes	0	0	0	0	0	0	0	0	1	0	0	-	1
Walking	Ioannidou	2017	Real world	yes	0	0	0	0	0	0	0	0	1	0	1	-	2
Walking	Jovancic	2006	HMD	yes	0	1	0	1	0	0	1	0	1	1	1	Top-down monitoring of pedestrians	6
Walking	King	2009	Real world	yes	1	0	0	0	0	0	0	0	1	0	0	-	2
Walking	Luo	2008	Real world	yes	0	0	0	0	0	0	0	0	0	1	0	Top-down influence on saccade behavior	1
Walking	Marigold	2007	Simulator	yes	0	0	1	0	1	1	1	0	1	1	1	Obstacle detection	7
Walking	Marigold	2008	Real world	no	0	1	1	1	1	0	1	1	1	1	1	Monitor environment and adjust steps	9
Walking	Miyasike-daSilva	2011	Real world	yes	0	0	0	0	0	0	0	0	1	1	1	Detection of handrail and control of limb movements	3
Walking	Miyasike-daSilva	2016	Real world	yes	0	1	1	1	1	1	1	1	1	1	1	Monitoring of stairs and controlling steps	10
Walking	Miyasike-daSilva	2019	Real world	no	0	1	1	0	1	1	1	0	1	1	1	Online control of stair locomotion	8
Walking	Murray	2014	Real world	yes	1	1	1	0	1	0	1	0	1	1	1	Provides egocentric information	8
Walking	Patla	1998	Real world	no	0	0	1	0	1	0	1	0	1	1	1	Fine-tuning of limb trajectory during obstacle avoidance	6
Walking	Timmis	2017	Real world	no	0	0	0	0	0	0	0	0	1	1	1	Path planning	3
Walking	Tong	2017	HMD	yes	1	0	0	0	0	0	0	0	1	1	0	Guide future eye-movements	3

Studies are sorted first, for the three domains and, within each domain, in alphabetical order of the first author’s surname. If a criterion in the 11 categories was met (see Table 2), the value for that category for that paper was set to 1. In the second to last column, we summarize how the paper discussed peripheral vision and its functionality (i.e., how it is used). In the last column, we display the sum of these binary values for every paper. Note that this is not a quality assessment of the paper, but rather a metric of the extent to which the paper focused on peripheral vision.

The criteria we formulated were met by varying subsets of studies from our total set (see Table 4). The columns “met” and “% met” show the absolute and relative number of papers that met each of the criteria mentioned in the “criteria” column. As an example, the aggregated data show that 25% of all included papers characterized visual capabilities or that 40% of the papers compared conditions with different attentional loads or demands. The highest value (83%) was observed for the criterion “discussions based on own criterion,” which we consider important because papers that do not meet this criterion only refer to papers on peripheral vision, rather than discussing it directly. The table also shows how each combination of two criteria was met by the set of studies. For example, of the 83% of the studies that fulfilled the criterion “discussions based on own criterion,” 62% also discussed a specific functionality of peripheral vision.

**Table 4:** Amount and percentages of papers meeting the 11 content criteria (columns 1–3) and percentages of papers within each criteria category meeting a second criterion (columns 4–14)

Criteria	Met	Percent met	Visual capabilities characterized	Predictions on peripheral-vision usage	Peripheral-vision manipulation	Attentional manipulation	Peripheral vision manipulation check	Compares foveal and peripheral vision	Compares with limited peripheral vision	Different attentional load/demands	Discussions based on own results	Functionalities discussed	Effects on actions discussed
Visual capabilities characterized	15	25.00	100.00	46.67	66.67	20.00	46.67	60.00	13.33	26.67	100.00	66.67	46.67
Predictions on peripheral-vision usage	19	31.67	36.84	100.00	73.68	42.11	57.89	52.63	31.58	47.37	89.47	63.16	68.42
Peripheral-vision manipulation	27	45.00	37.04	51.85	100.00	29.63	51.85	70.37	25.93	33.33	92.59	66.67	59.26
Attentional manipulation	21	35.00	14.29	38.10	38.10	100.00	28.57	33.33	19.05	85.71	66.67	52.38	28.57
Peripheral vision manipulation check	17	28.33	41.18	64.71	82.35	35.29	100.00	76.47	41.18	35.29	94.12	82.35	70.59
Comparison foveal and peripheral vision	21	35.00	42.86	47.62	90.48	33.33	61.90	100.00	19.05	38.10	95.24	76.19	61.90
Comparison with and without (or limited) peripheral vision	8	13.33	25.00	75.00	87.50	50.00	87.50	50.00	100.00	37.50	100.00	87.50	87.50
Differences attentional load/demands	24	40.00	16.67	37.50	37.50	75.00	25.00	33.33	12.50	100.00	66.67	41.67	20.83
Discussions based on own results	50	83.33	30.00	34.00	50.00	28.00	32.00	40.00	16.00	32.00	100.00	62.00	48.00
Functionalities discussed	36	60.00	27.78	33.33	50.00	30.56	38.89	44.44	19.44	27.78	86.11	100.00	63.89
Effects on actions discussed	25	41.67	28.00	52.00	64.00	24.00	48.00	52.00	28.00	20.00	96.00	92.00	100.00

In column 1, the 11 criteria are listed. In columns 2 and 3, the number and percentage of studies meeting the criteria are displayed, respectively. In columns 4–14, the studies that met each criterion are further characterized. Percentages below 100% in a given row show the percentages of papers that met one of the other criteria. As an example, 46.67% of the papers that characterized visual capabilities also made predictions on peripheral vision usage (first criteria line, column 5)

### Discussed functionalities

Table 5 shows a summary of the peripheral vision functionalities discussed in each study. In the last row, it can be seen that over the three research areas, the monitoring functionality (13/62) and the presaccadic preview functionality (10/62) were mentioned most. Also, walking studies mainly mentioned a monitoring functionality (7/19), and driving studies focused more on presaccadic preview functionality (8/37). In contrast, these functionalities were little mentioned in aviation, with the monitoring and action planning functionalities only mentioned once each. Overall, 23 studies did not mention a specific functionality, which should not be taken to mean that they ignored peripheral vision, merely that they did not focus on it particularly.

**Table 5:** Overview of the functionalities discussed in the included papers (literature sources in parentheses)

Discussed functionality	Aviation	Driving	Walking	All areas
Monitoring	1 (Brams et al., 2018^SA^)	4 (Doshi & Trivedi, 2012^MD^; Edquist et al., 2011^A,E^; Gaspar et al., 2016^MD,CL^; Kountouriotis et al., 2011)	7 (Feld & Plummer, 2019^MD^; Jovancevic et al., 2006^MD^; Marigold et al., 2007; Marigold & Patla^O^, 2008; Miyasike-daSilva et al., 2011; Miyasike-daSilva & McIlroy, 2016^MD^; Murray et al., 2014^O^)	12
Presaccadic preview	0	8 (Danno et al., 2011^ES^; Huestegge & Bröcker, 2016^AF^; Luoma, 1984; Mourant & Rockwell, 1970^E,MD^; Shahar et al., 2012^E^; Shinoda et al., 2001; Strayer et al., 2003^MD^; Underwood et al., 2003^E^)	2 (Luo et al., 2008; Tong et al., 2017)	10
Saccade/eccentricity costs	0	5 (Kountouriotis & Merat, 2016^MD^; Lamble et al., 1999^E, MD^; Lehtonen et al., 2018^E^; Seya et al., 2013^CL^; Summala et al., 1996^E,CL^)	0	5
Action planning	1 (Yu et al., 2014^SA^)	1 (Cooper et al., 2013^CL^)	4 (Berencsi et al., 2005^O^; Marigold & Patla^O^, 2008; Miyasike-daSilva et al., 2019^O^; Patla, 1998^O^)	6
Other	0	3 (Alberti et al., 2014^E^; Lehtonen et al., 2014^E^; Zhang et al., 2016^E,ES^)	2 (Bardy et al., 1999; Timmis et al., 2017^MD^)	5
None*	4 (Imbert et al., 2014; Kim et al., 2010^E^; Robinski & Stein^E^, 2013^E^; Schaudt et al., 2002^MD^)	15 (Beh & Hirst, 1999^MD^; Bian et al., 2010^MD,CL^; Briggs et al., 2016^MD,CL^; Crundall et al., 2002^E,MD^; Crundall et al., 2004^CL^; Harbluk et al., 2007^MD^; Janelle et al., 1999^ES^; Lin & Hsu, 2010^MD^; Mayeur et al., 2008^CL^; Patten et al., 2006^E^; Tsai et al., 2007^MD^; Underwood et al., 2005^A^; Victor et al., 2005^MD,CL^; Zhao et al., 2014^E^; Zwahlen, 1989)	4 (Cinelli et al., 2009; Hasanzadeh et al., 2018^SA^; Ioannidou et al., 2017^MD^; King et al., 2009^A^)	24
Sum	6	37	19	62

Two studies (Marigold & Patla, 2008; Mourant & Rockwell, 1970) mentioned two functionalities, so that the sum of functionalities is 62, although we only included 60 studies

* Studies mentioned in the “None” category did not explicitly mention a specific functionality. Some studies discussed a functionality between the lines. Please see text for these interpretations.

*Abbreviations in exponent notes.* E – Expertise, MD – multitasking and distraction, CL – cognitive load, A – age, AF – action before fixation, ES – emotions and stress, SA – situational awareness, O – occlusion

## Qualitative results

This review is informed by our understanding that peripheral vision is so central to many real-world tasks that its role passes unremarked. Yet, by looking to research in driving, walking, and aviation, we might gain insights into peripheral vision and how it supports complex tasks that we undertake outside the laboratory. With this in mind, our review and discussion section is structured in two parts. In the first, we consider how drivers, pedestrians, and pilots use peripheral vision; that is, what information it provides and the evidence for its often unacknowledged role. In the second part, we ask what impacts our ability to use peripheral vision while driving, walking, and flying planes, why we do not always use it if it provides useful information, and how our ability to do so is limited.

### How we use peripheral vision

Here, we will look across our three very different real-world tasks to learn what the broad commonalities are in terms of how pilots, drivers, and pedestrians use peripheral vision, and how the ways in which they do so overlap. To impose some organization on the question, we have divided it into three subcases. The first is how peripheral vision is used to monitor our surroundings, an inherent component of most, if not all, real-world tasks. The second is how we use peripheral vision to plan action, and the third is how peripheral vision informs eye movements. While we have done this to impose some structure on an otherwise-unruly body of literature, we must also point out that these three functionalities of peripheral vision are intrinsically interwoven, and considering one without the other is likely to be an exercise in incompleteness and frustration.

#### Monitoring the environment

The use of peripheral vision for generalized monitoring can take many forms; pilots or air-traffic controllers may monitor the periphery against the occurrence of instrument failure (Brams et al., 2018; Imbert et al., 2014) or monitor instruments, like the speed indicator, while gazing out the windscreen (Schaudt et al., 2002). Similarly, drivers can use cues (e.g., warning lights or other simple visual alerts) that appear in the periphery to tell them when it is safe to change lanes, and drivers in fact perform better with these peripherally presented cues than with cues presented at fixation (Doshi & Trivedi, 2012), perhaps because drivers expect hazards due to a lane-changing maneuver to appear in their periphery. More broadly, a driver’s understanding of their overall environment no doubt leads them to expect hazards, like cyclists, to be in some parts of the scene, such as being on the road rather than in an arbitrary location (Zwahlen, 1989).

How are we able to monitor for changes in our environment? Our knowledge about the environment and the predictability of changes in that environment likely plays a considerable role. Cockpit instruments or alert lights in a car, for example, remain at a fixed position, which helps us to peripherally monitor a limited region of the visual field and allocate resources to this region, rather than monitoring the entire visual field all the time.

When the environment is less predictable, a wider visual field must be monitored with peripheral vision. That this is possible can be seen in a study by Marigold et al. (2008), where pedestrians in the laboratory were quite capable of noticing stumbling blocks that suddenly appeared in their path, without looking down at them. Critically, their participants’ ability to react to this obstacle without fixating it shows that they must be using peripheral vision. In another study, pedestrians texting and walking inherently used peripheral vision to avoid collisions, since the cell phone occluded their central vision (Feld & Plummer, 2019; see other references on peripheral monitoring in Table 5, “Monitoring”).

#### Peripheral vision for action

**Fig. 3:**
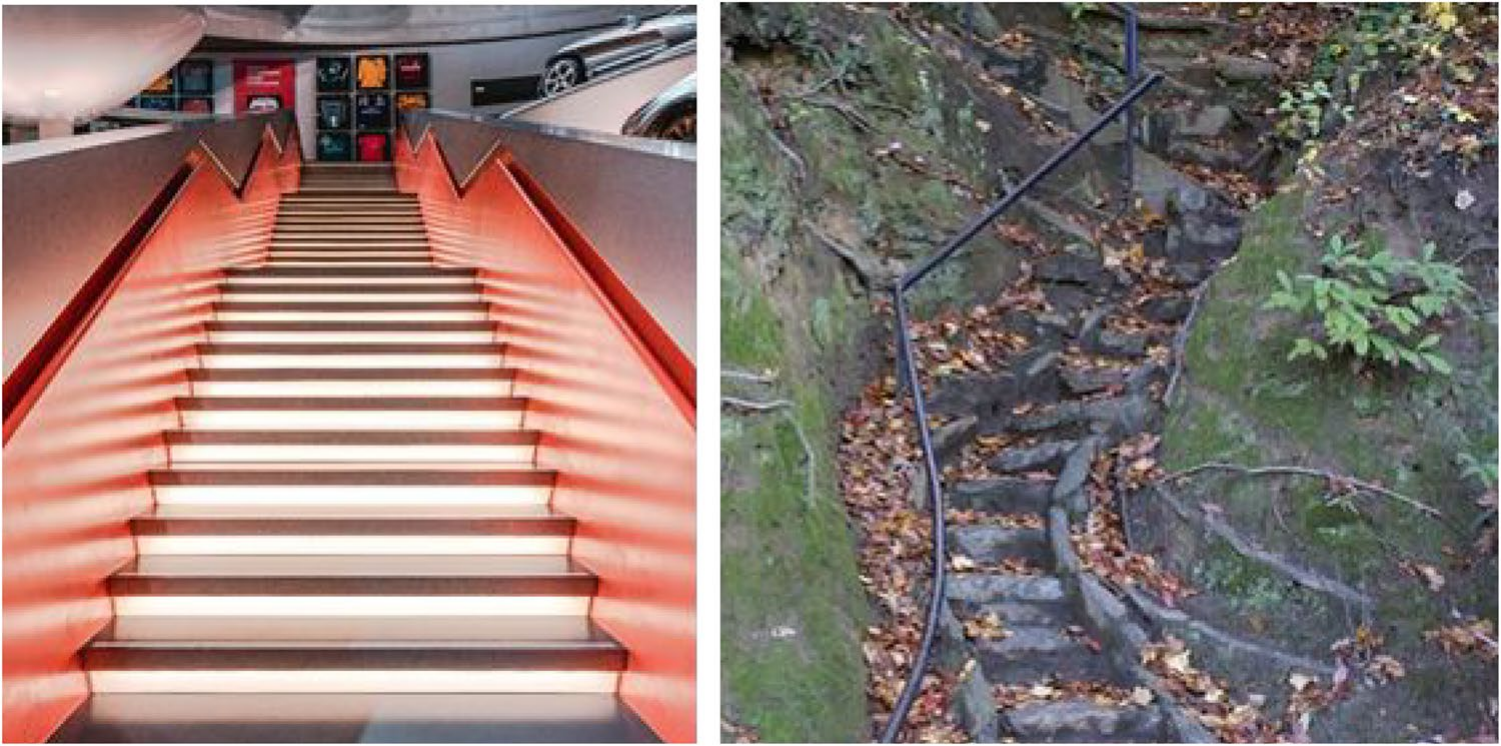
The left image (Sara Kurfeß, CC0 1.0) shows easy-to-walk stairs while the right image (taken by Greenville, SC Daily Photo, CC0 1.0) shows difficult stairs. The easy stairs are regular and can likely be walked using only peripheral vision. In contrast, the stairs on the right are very uneven and narrow (and are likely slippery due to the wet leaves on them). Their irregular nature will not be represented in sufficient detail with peripheral vision, requiring a pedestrian to look at each step as they ascend or descend them

People can also perform some actions while relying only on peripheral vision. Whether or not this is possible depends significantly on the environment. For example, when walking down a flight of stairs, we habitually fixate transitional steps, which define the point of change between a level surface and a staircase, but often rely on peripheral vision to provide enough information about intermediate steps (Miyasike-daSilva et al., 2011). On the other hand, some environments and some staircases (as shown on the right-hand side of Fig. 3) demand careful fixation of each step because they are neither level nor predictable. This makes ascending or descending such a staircase a much slower and more methodical process. Given a predictable environment, we have little trouble ascending a staircase using only peripheral vision (Miyasike-daSilva & McIlroy, 2016). If pedestrians are restricted from using peripheral vision by experimental manipulation – in particular, if they are unable to use the lower visual field (Marigold & Patla, 2008) – they behave much as when climbing an uneven staircase, that is, looking at each tread to plan a step (see also Miyasike-daSilva et al., 2019). On the other hand, restricting central vision (Murray et al., 2014) does not adversely impact stair-climbing behavior, although the lack of fine detail might prove problematic in less-predictable environments, and perhaps makes the transition between the stairs and a flat surface harder to navigate. It seems that climbing stairs is possible with peripheral vision only, but why do people not look at the stairs? Perhaps because they want to see the path ahead, avoiding collisions and planning their next steps, similar to how pedestrians change how far ahead they fixate as a function of the difficulty of the walking path (Matthis et al., 2018).

We can see a similar reliance on peripheral vision in drivers using the location of road markings in their periphery to help them center their vehicle in a given lane (Robertshaw & Wilkie, 2008). Their ability to do so suffers if the information is not available on both sides of the road (Kountouriotis et al., 2011). Small amounts of optic flow (local motion signal) can indicate a lane departure. One possibility is that this is the cue people use to stay in their lane. People are apparently not only capable of monitoring this in peripheral vision, but in fact do use peripheral vision for monitoring this simple, high-contrast motion cue. Similar cues from the edge of the sidewalk are probably at play when staying on a path while walking (Bardy et al., 1999; Cinelli et al., 2009; Patla, 1998) and to monitor posture (Berencsi et al., 2005).

Together, walkers use peripheral vision to guide their feet, drivers to stay in their lane, and pilots to localize and operate controls in the visual periphery (Yu et al., 2014). In all of these examples, the actor chooses a fixaton location that has a certain distance from the to-be-controlled movement. The fact that they are reacting to object changes without looking at them clearly indicates the use of peripheral vision. The open questions here are: in which situations can we (or even should we) rely on peripheral vision and when should we initiate an eye movement and rely on foveal vision (for all references on how peripheral vision is directly linked to actions, see Table 5, “Action planning”).

#### Peripheral vision and eye movements

A particular case where peripheral vision’s role has long been acknowledged is in planning eye movements. While patients with retinitis pigmentosa will learn to plan eye movements beyond the range of retinal input (Luo et al., 2008; Vargas-Martín & Peli, 2006), in the absence of this retinal degradation, peripheral vision is critical to planning saccades. But, what can we learn from the applied literature about what information is available to plan saccades?

#### Some tasks require fixation, and others do not

There is a range of tasks in the world that require foveation, that is, looking at a specific object or location because the task demands more detailed information than peripheral vision can provide (cf., grasping; Hayhoe et al., 2003). While peripheral vision can tell a participant in a driving simulator experiment that a sign has changed (say, from a stop sign to a yield sign), correctly identifying the sign requires it to be fixated (Shinoda et al., 2001; see also Tong et al., 2017, for similar results). The gap here between localization and identification speaks to the respective capabilities of peripheral and foveal vision. Peripheral vision is sufficient for drivers to notice that *something* has changed and to tell them where that change occurred, which is sufficient to plan a saccade, but fixating the changed object is often necessary to determine *identity* (Clayden et al., 2020; David et al., 2021; Motter & Simoni, 2008; Nuthmann, 2014).

#### People might use peripheral vision to avoid fixation of irrelevant information

One cannot interpret a failure to fixate a given object in the world as evidence that an observer is unaware of it. A particularly telling example here is that distracted drivers fail to look at roadside billboards, and fail to recognize them later; meanwhile distraction has less impact on their ability to operate their vehicle (Strayer et al., 2003). The information available from fixating the billboards is irrelevant to the core driving task, and the lack of fixation may indicate that the drivers recognized them as billboards and chose to ignore them. To our knowledge, this has yet to be tested empirically, but, among other approaches, an EEG study could reveal if billboards are suppressed in cortical areas when irrelevant to the driver’s task.

#### Fixation is not always needed for action

Assuming that a given object needs to be fixated in order to plan an action in response to it can be problematic, since any motor action in the world takes time to plan and execute. For example, if the car ahead of you suddenly stops, would you fixate it first, and only then step on your own brake pedal? A recent study shows that drivers respond *prior* to fixating the hazard (Huestegge & Bröcker, 2016), relying on peripheral vision to tell them where the hazard is, and prioritizing response. We can see a similar reliance on peripheral information when it comes to detecting a motorcycle rider overtaking another vehicle, where drivers use information from their side mirror in the periphery to provide a general sense of their environment and to time their response (Shahar et al., 2012). The tendency for some actions to precede shifts in gaze when it comes to real-world tasks is counterintuitive and often at odds with our introspections about where we look and when (Luoma, 1984). Besides peripheral information processing, it is important to use depth information (Greenwald & Knill), optic flow information (Warren & Hannon, 1988) and flow parsing (Fajen & Matthis, 2013; Matthis & Fajen, 2014) to succesfully navigate through an environment (all references that found actions before fixating a target received the exponent note “AF” in Table 5).

#### The tradeoffs to initiate a saccade (or not)

It takes time to saccade back to important information if you look away, which means there are tradeoffs in deciding whether or not to saccade. On the road, for example, quick responses have to be made in response to hazardous situations. In such scenarios, participants seem to take the costs of saccades into account and detect a hazard 200–400 ms *before* they fixate the hazard (Huestegge & Bröcker, 2016). If drivers are forced to look away from and back towards the road, for example, when the costs of saccades are artificially raised, their ability to drive safely suffers (Lehtonen et al., 2018). This effect scales with the amplitude of the necessary saccade, with nearer objects requiring shorter saccades and having reduced impacts on the driver’s overall understanding of their environment (Danno et al., 2011).

#### Saccade tradeoffs depend on expertise and situational awareness

Expertise and situational awareness influence how well we can use peripheral vision. Our ability to use peripheral vision instead of saccades is almost certainly a function of our expertise with a given situation (Lamble et al., 1999; Summala et al., 1996; Underwood et al., 2003) and our level of situational awareness for the situation as a whole (Hasanzadeh et al., 2018). We review expertise effects and effects of load and distraction in the next section (all references on eye movements and its costs can be found in Table 5, “Saccade/eccentricity costs”; all references on situation awareness received the exponent note “SA”).

### What impacts how we can use peripheral vision in real-world tasks?

Since we are not born knowing how to fly a plane, drive a car, or even walk, there is a vast amount of expertise we develop, and the literature shows that a component of our expertise is the ability to use peripheral vision when it is advantageous to do so. After discussing how expertise affects use of peripheral vision, we discuss how cognitive load, distraction and even certain emotional states reduce our ability to use peripheral vision, and what the consequences are.

#### The role of expertise

Becoming skilled at a real-world task like driving or flying a plane, or even a task as seemingly simple as walking, means developing perceptual expertise that supports our ability to complete these tasks. Our expertise affects how we use peripheral vision. Expert drivers look primarily at the road ahead (Summala et al., 1996), while novice drivers gaze about much more widely (Crundall et al., 1999; Mourant & Rockwell, 1970), suggesting that experts are better able to use peripheral information (Alberti et al., 2014). For that matter, novice drivers are slower, on the whole, to notice peripheral changes (Zhao et al., 2014), which implies that while the input is available to them, they have not yet learned to make sense of it (Patten et al., 2006). Alternatively, expert drivers might simply be better able to use executive control to maintain sustained attention to the more important information – the road ahead (Alberti et al., 2014). Particularly in the case of highway driving, most safety-critical information is in the road ahead, and choosing to focus on that area of the scene might provide all, or nearly all, the information the driver truly needs.

This pattern in which the impact of expertise is revealed by changes in gaze pattern can be seen beyond driving. When comparing where trainee and expert helicopter pilots look, trainee pilots used a broad search strategy similar to that used by novice drivers (Robinski & Stein, 2013). Skilled drivers, pilots, and pedestrians must learn to use optic flow cues to maintain heading and position, and novice pilots, even if they have been taught to look at the vanishing point, must learn to use the available cues (Kim et al., 2010). In a similar vein, expert drivers fixate further ahead than do novice drivers, allowing them to anticipate, for example, turns in the road (Lehtonen et al., 2014; also Mars & Navarro, 2012).

Another reason for the change in gaze patterns may be that experts can better make use of imperfect information available in peripheral vision. Skilled drivers, with knowledge of how their vehicle and pedestrians tend to move, often need only a glance, if that, at an oncoming pedestrian to avoid a collision (Jovancevic et al., 2006). A pilot or driver’s ability to push a button or use a control without looking away from the windshield reflects a deep understanding and detailed mental model of their proximate environment, that is, the cab of the plane or vehicle (Yu et al., 2014). The predictability of a control panel affords this understanding, since buttons and gauges can be expected to stay in the same location, but in addition pilots must develop the perceptual expertise to interact with controls without looking at them directly (Yu et al., 2014).

Expertise, of course, interacts with age. Across our lifespan, we walk, drive, and fly for decades, but age might diminish our capacity to benefit from our expertise. Furthermore, the ability to acquire peripheral information likely declines with age (Owsley, 2011; Scialfa et al., 2013). Older drivers are not, however, always worse than younger drivers; often, they can detect as many road hazards as their younger compatriots (Underwood et al., 2005), and they can detect transients in their peripheral field of view while driving (Ward et al., 2018), but they are prone to more perceptual and motor errors, like steering their car less carefully (Edquist et al., 2011) or greater steering variability in following a lead vehicle (Ward et al., 2018). Experience could almost be said to breed a certain contempt for foveal vision; in a locomotion study, where participants would need to grab a handrail, older participants were less likely to fixate it on entering the space and less likely to grab it when they needed to (King et al., 2009). This, then, illustrates just how tricky the question of expertise is in the context of peripheral vision, and why it is worth considering as an evolution across the lifespan, rather than simple progress towards a peak (all references that link peripheral vision usage to expertise effects received exponent note “E” and those on age an “A” in Table 5).

#### Multitasking and distraction

Experts may see driving as one complex task – driving itself becomes quite automatic for them – while novices might understand driving as number of linked tasks requiring focus and attention, like steering the car while monitoring the environment for pedestrians, other vehicles, and road signs. Multitasking is inherent in these situations; for example, while driving, one must maintain awareness of the environment, control the brake and accelerator pedals, and maintain steering input. Visual perception studies, on the other hand, typically only explicitly introduce multitasking to study the effects of attention. The impacts of multitasking are often described in terms of the dangers of distraction (Strayer et al., 2019), and while these dangers are very real, our question here is what happens to someone’s ability to use and benefit from peripheral vision when they are multitasking, rather than the perils of distraction itself.

Distraction sometimes causes drivers to take their eyes off the road; this inherently causes them to be less aware of their operating environment, as it puts driving-relevant information into the periphery or outside of the field of view. However, distraction can cause problems even when the distracting task does not take the driver’s eyes off the road. In fact, auditory monitoring and driving-irrelevant visual detection tasks can produce similar effects: distracted drivers appear to rely more on peripheral vision for lane-keeping, but are less able to process and react to the information that peripheral vision provides (Gaspar et al., 2016; Lin & Hsu, 2010). On the other hand, Kountouriotis and Merat (2016) found that visual distractions caused more deviations in vehicle position than non-visual, though performance improved if one had a lead vehicle to follow. Distraction can also impact drivers’ ability to maintain fine control (Strayer & Johnston, 2001). Drivers performing an audioverbal arithmetic task gaze more at the road ahead, but are slower to react to changes in the environment than without the additional cognitive load (Harbluk et al., 2007; Tsai et al., 2007; Victor et al., 2005).

On the other hand, drivers appear to some extent to compensate for slower reaction times by changing their following distance or reducing their speed (Haigney et al., 2000). Similarly, in studies of distracted walking, for example due to texting, pedestrians slow down and remain able to navigate safely (Timmis et al., 2017). Even when climbing stairs while texting, participants are only moderately slower (20%), yet they can walk up the stairs without incident (Ioannidou et al., 2017). In walking and driving, we can see evidence for participants using peripheral information at a diminished but useful level even when distracted and looking away. Any difference between the safety of distracted walking and that of distracted driving may simply arise from the difference in how quickly one must react in order to be safe (largely due to differences in the speed of travel), rather than due to a fundamental difference in visual processing under high load conditions.

The impact of distraction depends greatly on task, and in particular participants seem to make a distinction between driving-relevant tasks and more irrelevant distractions. Cognitive load can greatly affect the detection of driving-irrelevant events (like a driving-irrelevant light flashing on the dashboard), and does so more in the upper than in the lower visual field (Seya et al., 2013). However, it is unclear whether this represents a degradation in peripheral vision with load, or a rational tradeoff between critical driving tasks and other tasks (as shown in studies where load has been imposed by such a driving-orthogonal task; Crundall et al., 2002; see also Bian et al., 2010; Gaspar et al., 2016; Mayeur et al., 2008). In driving, particularly, distraction impairs the ability to report irrelevant stimuli, suggesting that distraction might lead to tradeoffs in effort between two driving-irrelevant tasks – performing the nominally distracting task (e.g., using a cell phone) versus processing a less-relevant light on the dashboard or billboards on the roadside. Additional cognitive load can certainly impact observers’ performance, but the story may be complex because of compensatory behavior or tradeoffs between tasks. The pattern of eye movements can also be affected by distraction or multitasking. Distraction causes increased reliance on peripheral vision not only because drivers fixate on the distracting task, for example the texting app on their phone (Harbluk et al., 2007; Strayer et al., 2003), but because cognitive load can cause them to move their eyes differently (Briggs et al., 2016; Summala et al., 1996; Victor et al., 2005). Cognitive load can lead to drivers limiting their fixations to a smaller region of the visual field (Miura, 1986; Recarte & Nunes, 2003; Reimer et al., 2012), and this change cause ambiguity about whether distraction directly causes poorer performance, or does so indirectly by changing fixations. One can explicitly test the effects of gaze patterns as opposed to cognitive load per se by forcing drivers to maintain a particular fixation pattern and separately varying cognitive load. Using such an approach, Cooper et al. (2013) demonstrated that making eye movements over a narrow versus a wide range on the forward roadway had no effect on performance, but increasing cognitive load paradoxically led to better performance on the lane-keeping task, pointing to the complexities here.

One might think of a person’s internal state as a different sort of distraction. Angry drivers, for example, behave much like distracted drivers, and are less aware of their surroundings (Zhang et al., 2016). Anxious drivers, like those in a new driving environment or who are simply predisposed to worrying about their safety and that of everyone around them, also have difficulty in using peripheral information (Janelle et al., 1999). Overall stress has similar effects; when stressed, drivers do not look at objects in the periphery even when they need to, and are slower to respond to hazards (Danno et al., 2011). The results of these manipulations could be interpreted as tunnel vision, where drivers are *unable* to perceive beyond a certain spatial extent around fixation. Tunnel vision is often observed if the task requires a speeded response and includes foveal load (Ringer et al., 2016; L. J. Williams, 1985, 1988, 1989), which is the case in many of the included studies. However, given results questioning whether high cognitive load really leads to tunnel vision (Gaspar et al., 2016; B. Wolfe et al., 2019), a better hypothesis may be that certain emotional states (and other factors, like increased cognitive load) make it more difficult to perceive peripheral information, rather than impossible. Even something as seemingly mundane as loud music can have similar impacts on how drivers can use peripheral vision; it diminishes their ability to report peripheral events in a timely manner, while, counterintuitively, facilitating detection of central targets (Beh & Hirst, 1999). However, using a different form of auditory distraction, Briggs et al. (2016) showed worse hazard detection with greater cognitive load, independent of eccentricity of the hazard (see Table 5 for all references that link peripheral vision usage to multitasking and distraction – exponent note “MD,” to cognitive load – exponent note “CL,” and to emotions and stress – exponent note “ES”).

## General discussion

This review has shown that natural tasks, with their time constraints, more complex stimuli, and richer measures of performance, reveal new insights about how we use peripheral vision. For example, we use it during multitasking (many real-world tasks require at least dual tasking) and to guide our actions and eye movements. We identified tasks that people can solve without fixating task-relevant information – and our ability to do this clearly points to the use of peripheral vision to perform the task. Nonetheless, when using peripheral vision, performance can be affected by factors like our knowledge of the task, age, distraction, or the relative importance of multiple tasks. Therefore, it is essential to remember that there are always tradeoffs in deciding whether to use peripheral vision or eye-movements (foveal vision). To better understand these tradeoffs and to point to where research might go in the future, we will now integrate our review of these applied literatures with what is known in the context of sport and vision sciences. In addition, we will suggest three new approaches to research, drawn from this work, that might help further illuminate our understanding of peripheral vision more generally.

### Integrating peripheral vision findings across disciplines

Peripheral vision is used for monitoring the environment; a functionality reported in driving, walking, and aviation as well as in sport and vision science. The forms of monitoring, however, may be subtly different, particularly when comparing vision science to the more applied fields. A pilot using peripheral vision to monitor a peripheral gauge, or a driver navigating the road while noticing a motorcycle in the side mirror may be doing a gist-like scene-perception task (Larson & Loschky, 2009; Loschky et al., 2007; Oliva, 2005; Rousselet et al., 2005), for which they draw information from a sizeable region centered on their point of gaze (Mackworth & Morandi, 1967), while simultaneously monitoring known peripheral locations. Peripheral vision may rely upon simple low-level saliency to detect hazards or obstacles (Crundall et al., 2003), but it remains an open question whether this wide field of view monitoring additionally relies on more complex recognition processes like gist or event identification. In sports, athletes need to use their wide field of view, for example to monitor opponents and teammates (Vater et al., 2020).

A major difference between applied and basic science seems that monitoring is necessary but often not considered as a conscious task in the applied domains, compared to the explicit tasks common in vision science (as discussed in Vater et al., 2017a, 2017b). In sum, the diverse cases of monitoring that exist outside the laboratory suggest that we are almost always doing multiple tasks at once, without being aware we are doing some of them, because the world is too complex and dynamic to do otherwise.

While peripheral vision is, of course, essential in many cases to plan saccades (Deubel & Schneider, 1996; Kowler et al., 1995), it is merely one special case of what we use peripheral vision for more broadly. We can, to some degree, detect hazards, road signs, and obstacles with peripheral vision, and use this info to guide a saccade if needed. However, a number of factors make it difficult to assess saccade planning. From vision science, we know that reducing information in the periphery (e.g., by removing information from peripheral vision with image filtering or adding noise) may reduce the likelihood of saccades to these less informative locations (Cajar et al., 2016; Nuthmann, 2014). In our review, we note that it is also a question of task demands, what the observer knows about the environment, and the tradeoffs involved in making or withholding a saccade. Making a saccade always puts previously foveated information in the periphery, which can have its costs. For example, looking away from the road ahead can result in a collision when the car ahead brakes, but the driver fails to perceive that braking in time (foveating the car would have been better). In sports, looking away from the opponent in martial arts can result in losing a bout when the punch or kick is seen too late (Hausegger et al., 2019). In both examples, the task must be solved under time pressure, and under these circumstances, the observer must account for the potential information they might acquire by moving their eyes, but also the information they would lose while the saccade was in progress. If researchers do not properly address factors like time pressure and situational contexts, one could, for instance, erroneously reason that driving experts know less about peripheral hazards than novice drivers, because experts rarely fixate hazards.

Our review, additionally, provides key insights into the factors that impact our ability to use peripheral information, including knowledge, aging, distraction, and emotional state. That greater *knowledge* or expertise leads to better visual performance is, of course, an accepted fact. However, at least in basic vision science, the prior knowledge often takes the form of reducing the number of likely target locations in a search task, or reducing the set of possible objects in an object recognition task. For example, prior knowledge aids monitoring not only in driving studies but also in sports (M. Williams & Davids, 1995) as well as in basic vision science (Castelhano & Heaven, 2011; Draschkow & Võ, 2017; Tsai et al., 2007). In vision science, it is understood that our knowledge about scene context helps to identify peripheral objects, at least in part by narrowing down the possible objects to those likely to occur in the scene (Wijntjes & Rosenholtz, 2018). In sport science, experts are better able to monitor the movements of other players (Vater et al., 2019), which may be due to additional knowledge about the likelihood of certain movements. However, while applied vision shows similar effects, like the ability to use peripheral vision to interact with buttons or monitor alerts at known locations, knowledge can also impact use of peripheral vision in a somewhat different way. In some real-life situations, people can quickly acquire enough information from a single glance at an object to enable them to then rely only on peripheral vision. For example, a single glance at a pedestrian and a driver can, thereafter, monitor the pedestrian well enough to avoid a collision (cf., Eckstein et al., 2006; Torralba et al., 2006). With a glance to gather knowledge about the stairs, one can continue up them without further need to fixate each riser. Route familiarity induces drivers to use peripheral vision more than they would on an unfamiliar route (Mourant & Rockwell, 1970). It is as if one can become an “expert” about a particular location or situation, sometimes from a mere glance, and then, as needed, fill in the information not available to peripheral vision. If so, one might expect to observe more effects of expertise and knowledge in peripheral vision than in more foveal tasks. To put it simply, knowledge may improve the utility of limited peripheral information. However, *age* is closely intertwined with expertise because the older participants are, the more knowledge they have (theoretically) acquired. Yet, from fundamental research, we know that contrast and acuity decline with age (Owsley, 2011). That means, especially for applied research, that declines in visual capability and expertise effects need to be separated, rendering the question of expertise more complicated.

*Distraction* has been long known to have adverse perceptual impacts, as shown in inattentional blindness (Mack & Rock, 1998; Wood & Simons, 2019) and dual-task experiments (Rosenholtz et al., 2012; VanRullen et al., 2004). In real-world tasks as well as in basic science research on tunnel vision, distraction changes fixation patterns, both when there is a secondary visual task and also simply indirectly due to load (Gaspar et al., 2016; Ringer et al., 2016; Ward et al., 2018). How distraction affects the use of peripheral vision in sports has yet to be examined. It can, however, be expected that distraction is a factor, for example, when a basketball player is preparing to free-throw a ball, the members of the crowd supporting the opposing team might intentionally move and make noise to try to distract them. Furthermore, *emotions* and their impact on perception are well studied in sports, and the effects of stress and anxiety on performance are known to impact decision times and gaze behavior (Vater, Roca, & Williams, 2016b) and especially the processing efficiency of foveated information (Vine et al., 2013). Vision science has examined questions of *valence*, i.e., the impact of the stimulus attractiveness or averseness on performance of visual tasks (e.g., happy, sad, or scary stimuli impact reaction times or lead to distraction), rather than the impact of emotional states (Bugg, 2015). The result that emotions cause people to miss peripheral targets, particularly when they are task-irrelevant, may suggest a tradeoff between relevant and irrelevant information, under “load” from one’s emotional state, analogous to the impact of more general cognitive load (Engström et al., 2017).

### Three recommendations for future peripheral vision research

Our goal here is to propose three potential avenues for future research, drawing from this review: First, probing the contribution of various portions of the visual field to determine their role in particular tasks, and to confirm or refute our view of peripheral vision’s role in these tasks. Second, to use eye tracking in a new way, and rather than asking where participants look, ask where they do not, since the absence of a gaze to a certain location does not mean the participant has no information. Finally, we suggest looking at cases where participants are or are not permitted to look at particular locations, to determine whether their informational needs can only be met by saccades and subsequent fixation, or if peripheral vision can serve their needs. These approaches draw from techniques (e.g., gaze-contingency paradigms, as pioneered by McConkie & Rayner, 1975) used across basic and applied research, but will provide answers to key questions at the intersection of real-world tasks, peripheral vision, and saccades.

The first line of research focuses on occluding portions of the field of view, or vision entirely (it may be possible that vision is not needed at all), to investigate changes in performance and see if the occluded region of the visual field made a meaningful contribution to the task at hand (for an early study on walking with very low vision and limited peripheral vision see Pelli, 1987; all references that used occlusion methods in the set of included studies received the exponent note “O” in Table 5). That it is possible to drive a car even without vision – at least for some seconds – has been shown in self-paced occlusion studies on real roads (cf., Senders et al., 1967). This research shows that especially experienced drivers sometimes do not need vision at all times to steer a car (for a recent review, see Kujala et al., 2021). Therefore, it is important to figure out when and how peripheral vision is used. One way to do this is with gaze-contingent paradigms, which is common in vision science and in some applied laboratory studies (e.g., see Ryu et al., 2013; Ryu et al., 2015), and allow stimuli to be manipulated based on where the observer is looking at any given time. This paradigm could also be useful to investigate peripheral preview capabilities (all references discussing the preview functionality can be found in in Table 5, “Presaccadic preview”). One could also manipulate the usefulness of information on the saccade target during or immediately prior to the saccade. It is hypothesized that if peripheral information is used, then the fixation duration on the target will be less when the information remains the same, but longer when it is changed (as it needs to be updated with foveal vision). In on-road studies, where such stimulus control is impractical, participants could be instructed where to look (and control that with eye-tracking or tasks which require fixation; e.g., Wolfe et al., 2019), which has been done in some driving studies (Lehtonen et al., 2014; Lehtonen et al., 2018). By doing so, it becomes possible to control the eccentricity of events and to determine how task performance and reaction time change accordingly; that is, the penalties that occur when an observer must rely on peripheral vision.

The other two revealing lines of research use precise eye tracking in conjunction with motor responses that reveal what information the participant requires for a particular task. One variant of this would be to ask whether participants are using information they are *not* fixating, suggesting a reliance on peripheral vision, to complete specific actions. This might be done relatively easily, since applied research often lets participants freely view their environment while monitoring gaze position (cf., Peißl et al., 2018, for a review on eye tracking in aviation; also Ziv, 2017). For example, if participants do not fixate an obstacle, but step over it, they must have used peripheral vision to do so (Marigold et al., 2007; see Marigold, 2008, for a review). Similarly, in driving, if a driver began to steer away from an obstacle before fixating it, this could have only been based on peripheral information. It should, however, be noted that it can be is easy to misuse and misinterpret eye-tracking data (B. Wolfe et al., 2020), since it is impossible to be certain that participants are actually using the information they are fixating (e.g., looked-but-failed-to-see errors, Herslund & Jørgensen, 2003).

Finally, one can reason about what peripheral vision might be used for by making use of models of the information available across the field of view and across a saccade. Vision science has made considerable progress on modeling and visualizing the information preserved in peripheral vision (Balas et al., 2009; Deza et al., 2018; Doerig et al., 2019; Freeman & Simoncelli, 2011; Rosenholtz, Huang, Raj, et al., 2012). These models, and work inspired by them, may help experimenters identify relevant information that does or does not survive peripherally as a function of eccentricity, helping us understand why we may or may not saccade to and fixate an object.

## Summary

Using peripheral vision is intrinsic to many real-life tasks, like driving, walking, and aviation, and its role is acknowledged in sport science and well investigated in vision science, but no review has tried to draw together all of these very different threads. Here, we have done so, showing commonalities across a range of different tasks in very different settings, reflecting a global functionality for peripheral vision, anchored in monitoring and saccade planning, but that defies simple classification, since these functionalities are susceptible to interference from distraction, multitasking, and other factors. We then go on to draw on all of these very different elements to propose avenues for future research, including manipulating what visual information is available, investigating assumptions about what tasks require foveal information, and examining when and why we look where we do in real-world tasks, based on our informational needs. Peripheral vision is the sea we all swim in, from basic research in the laboratory to practitioners solving problems in the field, and by understanding how and why we use it, and when and why we do not, we can better understand its capabilities and limitations, and better explain human behavior.

## Data Availability

Table 3 is provided as an excel document on the Open Science Framework (https://osf.io/vea5r/?view_only=ba8597fef6514be68082d9e878fff5d2). The review was not pre registered.
